# Changes in serum pain mediators, stress response, inflammatory markers, and coagulation function in lumbar disc herniation patients treated by musculoskeletal ultrasound-guided needle-knife

**DOI:** 10.5937/jomb0-59930

**Published:** 2026-01-28

**Authors:** Jiaqi Wu, Ming Chen, Wenlong Liu, Jiaming Jiang, Sihan Chen, Tingyu Liu, Xiaoyan Liu

**Affiliations:** 1 Jingjiang People's Hospital Affiliated to Yangzhou University, Department of Rehabilitation Medicine, Taizhou, China; 2 Jingjiang People's Hospital Affiliated to Yangzhou University, Department of Ultrasound Diagnosis, Taizhou, China

**Keywords:** lumbar disc herniation, musculoskeletal ultrasound, needle-knife therapy, stress response, inflammation, lumbalna disk hernijacija, mišićno-skeletni ultrazvuk, terapija iglom-nožem, odgovor na stres, upala

## Abstract

**Background:**

To investigate the impacts of musculoskeletal ultrasound (MSK-US)-guided needle-knife on serum pain mediators, stress response, inflammatory markers, and coagulation function in lumbar disc herniation (LDH) patients.

**Methods:**

In this prospective cohort study, we recruited 100 LDH patients admitted between February 2024 and January 2025 and assigned them to an MSK-US group (52 cases undergoing MSK-US-guided needle-knife) and a conventional group (48 cases receiving conventional needle-knife). Peripheral venous blood was collected before and within 2 hours postoperatively for IL-6, TNF-a, SP 5-HT, and hs-CRP measurements by ELISA, Cor, E, and NE determination using an automatic biochemical analyzer, b-EP detection by chemiluminescent immunoassay, and APTT, PT, FIB, and D-D quantification with a coagulation analyzer. Dynamic changes in these biomarkers were analyzed by betweenand within-group comparisons.

**Results:**

Both groups demonstrated postoperative reductions in SP and 5-HT and an elevation in b-EP (P&lt; 0.05), though no marked inter-group differences were noted (P&gt; 0.05). The MSK-US group showed lower postoperative levels of Cor, E, NE, IL-6, hs-CRP D-D, and FIB than the conventional group (P&lt; 0.05), suggesting MSK-US's ability to reduce inflammation, alleviate stress reaction, and lower hyperfibrinolysis risk.

**Conclusions:**

MSK-US-guided needle-knife is effective in mitigating perioperative stress response and systemic inflammation in LDH patients while reducing the risk of coagulation disorders and providing a biomarker-driven framework for personalized postoperative management.

## Abstract (sr)

**Uvod:**

Cilj je ispitivanje uticaja igle-noža vođenog mišićno-skeletnim ultrazvukom (MSK-US) na medijatore bola u serumu, odgovor na stres, inflamatorne markere i koagulacionu funkciju kod pacijenata sa lumbalnom disk hernijacijom (LDH).

**Metode:**

U ovoj prospektivnoj kohortnoj studiji, regrutovali smo 100 pacijenata sa LDH primljenih između februara 2024. i januara 2025. godine i svrstali ih u MSK-UZ grupu (52 slučaja kojima je primenjena MSK-UZ vođena igla-nož) i konvencionalnu grupu (48 slučajeva kojima je primenjena konvencionalna igla-nož). Periferna venska krv je prikupljena pre i u roku od 2 sata nakon operacije za merenja IL-6, TNF-aa, SP 5-HT i hs-CRP pomoću ELISA testa, određivanje kortikosteroida, estradiola i neurona pomoću automatskog biohemijskog analizatora, detekciju b-EP hemiluminiscentnim imunotestom i kvantifikaciju APTT, PT, FIB i D-D pomoću analizatora koagulacije. Dinamičke promene ovih biomarkera analizirane su poređenjima između i unutar grupa.

**Rezultati:**

Obe grupe su pokazale postoperativno smanjenje SP i 5-HT i povišenje b-EP (P&lt; 0,05), iako nisu primećene značajne razlike između grupa (P&gt; 0,05). MSK-US grupa je pokazala niže postoperativne nivoe Cor, E, NE, IL-6, hs-CRP D-D i FIB u odnosu na konvencionalnu grupu (P&lt; 0,05), što sugeriše sposobnost MSK-US da smanji upalu, ublaži reakciju na stres i smanji rizik od hiperfibrinolize.

**Zaključak:**

MSK-US vođena igla-nož je efikasna u ublažavanju perioperativnog stresnog odgovora i sistemske upale kod pacijenata sa LDH, istovremeno smanjujući rizik od poremećaja koagulacije i pružajući okvir za personalizovano postoperativno lečenje zasnovano na biomarkerima.

## Introduction

Lumbar disc herniation (LDH) is a prevalent degenerative disorder in orthopedic practice, affecting around 15.4% of the global population and significantly impairing patients' quality of life while imposing grave socioeconomic burdens [1]. Its pathogenesis is indicated to involve complex interactions among intervertebral disc degeneration, mechanical compression, and inflammation. Notably, dysregulation of pain mediators, stress hormones, and proinflammatory cytokines has been identified as a crucial factor contributing to pain exacerbation and neurological dysfunction [2] [3] [4].

As medical technology advances, minimally invasive procedures have emerged as primary treatment options for LDH [5]. Among various minimally invasive approaches, needle-knife demonstrates distinctive advantages through its ability to release tissue adhesions and restore biomechanical balance [6]. While musculoskeletal ultrasound (MSK-US) enables real-time visualization of paraspinal soft tissue structures and facilitates precise needle-knife guidance, the underlying therapeutic mechanisms need further clarification. Current studies predominantly concentrate on clinical symptom improvement [7], leaving significant gaps in systematic investigations of treatment-induced changes in serum pain mediators, stress response modulation, and inflammatory cascade regulation. Furthermore, surgical interventions necessitate careful monitoring of coagulation function, as operation-induced stress responses often impair hemostatic mechanisms, potentially leading to postoperative complications like hemorrhage and infection [8].

This study innovatively integrates MSK-US guidance technology with needle-knife therapy. Through serial blood sampling at multiple time points, we systematically evaluate serum pain mediators, stress response markers, and inflammatory markers while monitoring changes in patients' coagulation function. The research aims to address four key scientific questions: ① whether visual guidance enhances analgesic efficacy by improving targeting precision during intervention; ② whether pain relief correlates with specific modulation of central sensitization-related mediators; ③ whether temporal patterns in stress response and inflammatory markers can serve as predictors for long-term clinical outcomes; and ④ how MSK-US-guided minimally invasive procedures affect coagulation parameters. The findings are expected to contribute theoretical foundations for optimizing needle-knife precision while potentially establishing a biomarker-based personalized efficacy assessment system. Furthermore, this investigation may provide novel insights into the mechanistic understanding of minimally invasive treatments for lumbar degenerative diseases.

## Materials and methods

### Research design

This study adopts a prospective cohort design conducted at a single center. The target population comprises patients diagnosed with LDH who presented at our hospital between February 2024 and January 2025. Sample size estimation was based on the effect size (Cohen's d= 0.8) of changes in serum pain mediators (e.g., substance F) SP) before and after treatment, as observed in our preliminary data involving 20 patients (unpublished observations). With α = 0.05 (two-tailed) and β = 0.2 (80% power), the required sample size was calculated using a two-independent-sample t-test, yielding at least 42 participants per group. To account for a potential 10% dropout rate, the final recruitment target was set to 47 participants per group. This study has been approved by the ethics committee of our hospital, and all subjects signed informed consent.

### Inclusion and exclusion criteria

Inclusion criteria: (1) LDH diagnosis based on [9]: ① low back pain accompanied by radiating pain in the lower limbs; ② positive straight leg raise test (≤60°); ③ confirmation via lumbar Magnetic Resonance Imaging (MRI) or Computed Tomography (CT) showing single-segment herniation (L4-L5 or L5-S1, the most commonly affected segments in LDH) with corresponding nerve root compression; ④ symptom duration of 1-12 months with inadequate response to conservative treatments (e.g., non-steroidal anti-inflammatory drugs, physiotherapy); (2) Age 18-65 years, any gender: (3) Willingness to provide informed consent and complete the 3-month follow-up.

Exclusion Criteria: (1) Severe lumbar spinal stenosis, spondylolisthesis (grade II or higher), spinal tumors, or infections; (2) Lumbar surgery, epidural injections, or nerve blocks within the past month; (3) Bleeding disorders or long-term anticoagulant use; (4) Pregnancy, lactation, or severe dysfunction of major organs (heart, liver, kidneys); (5) Psychiatric conditions impairing compliance with assessments.

### Grouping and intervention methods

Following inclusion and exclusion screening, 100 LDH patients were finally enrolled in the study. Participants were randomly assigned using a computer-generated random number sequence to either the MSK-US group (52 cases undergoing MSK-US-guided needle-knife) or the conventional group (48 cases receiving conventional needle-knife). The procedure was performed using a LOGIQ E9 color Doppler ultrasound diagnostic system (GE Healthcare) equipped with a high-frequency linear array transducer (7-15 MHz). The patient was placed in a prone position, and the affected lumbar intervertebral space (L4-L5 or L5-S1) was identified via ultrasound scanning. The examination assessed the location and size of the intervertebral disc protrusion as well as the nerve root compression degree. The target area— between the medial edge of the facet joint and the outer notch of the lamina—was marked as the needle insertion point. After local anesthesia with 2% lidocaine, a Hanzhang I No. 4 needle-knife (diameter: 0.83-5 mm, length: 4 cm) was inserted under realtime ultrasound guidance. The needle tip was then advanced to the outer notch of the lamina or the articular capsule of the facet joint. Subsequently, longitudinal or transverse cutting and release were performed (2-3 incisions), with the depth carefully controlled at 3-5 mm. Upon needle withdrawal, manual pressure was applied to achieve hemostasis, followed by coverage of the puncture site with an adhesive bandage. The intervention was administered once per week, for a total of three sessions.

Another cohort of 48 patients underwent standard needle-knife treatment (conventional group) following the same protocol. The procedure was performed without ultrasound guidance, relying solely on anatomical landmarks (determined by palpation of spinous processes and iliac crests) for needle insertion point localization. The needle trajectory, depth of insertion (typically aiming for bony contact at the lamina or facet joint), and extent of tissue release (typically 2-3 longitudinal or transverse cutting motions) were determined based solely on the surgeon's tactile feedback and clinical expertise.

Quality control: All procedures were conducted by the same surgical team, with each member possessing over five years of experience in either conventional or ultrasound-assisted techniques. Prior to the study, all personnel underwent standardized training and evaluation. Additionally, both participants and data collectors were blinded to the patient allocation.

### Sample collection and testing

Preoperative (baseline) and postoperative (within 2 hours) peripheral venous blood samples were collected using Ethylene diamine tetraacetic acid dipotassium salt anticoagulant vacuum tubes. Immediately after collection, the tubes were gently inverted 5-8 times to ensure proper mixing and prevent coagulation or hemolysis. The samples were then allowed to stand at room temperature for 30 minutes. Subsequently, centrifugation was performed at 3000 Xg (15 cm rotor radius) for 10 minutes. The supernatant serum was carefully transferred into sterile EP tubes and aliquoted into three 100 μL portions to minimize freeze-thaw cycles. All serum samples were stored at -80 until analysis. Prior to testing, the samples were thawed gradually at room temperature.

Enzyme-Linked Immunosorbent Assay (ELISA) detection of Interleukin-6 (IL-6), Tumor necrosis factor-α (TNF-α), Substance P (SP), 5-hydroxytryptamine (5-HT), and hypersensitive-C reactive protein (hs-CRP): The Quantikine ELISA kit (R&D Systems) was employed for analysis. Each well was added with 100 μL of the sample, followed by a 2-hour room-temperature incubation. After washing, 100 μL of biotin-conjugated secondary antibody was added and incubated for 1 hour. Subsequently, 100 μL of HRP substrate solution was introduced and kept in the dark for 30 minutes. The reaction was stopped with 50 μL of termination solution. Absorbance at 450 nm was measured using a microplate reader, and sample concentrations were determined by fitting a four-parameter logistic regression standard curve. For quality control, high- and low-concentration serum controls (Bio-Rad Immunoassay Plus Control) were included on each plate, with a coefficient of variation (CV) < 15%.

Automated biochemical analysis of Cortisol (Cor), Epinephrine (E), and Norepinephrine (NE): Measurements were performed on a Roche Cobas e601 analyzer, with samples processed directly by the system. Quality control was ensured using Bio-Rad Lyphochek Immunoassay Plus Control, maintaining a CV < 10%.

Chemiluminescence immunoassay for β-Endorphin (β-EP) detection: β-EP levels were measured using an automated chemiluminescence immunoassay (AutoLumo A1800) with the Siemens ADVIA Centaur XP β-EP kit. The procedure involved mixing 100 μL of serum with 50 μL of labeled antigen, followed by a 9-minute incubation at 37. Subsequently, 100 μL of paramagnetic particles coated with solid-phase antibodies were added, and the mixture was incubated for another 9 minutes. After magnetic separation to remove unbound components, an excitation solution containing ruthenium tripyridine was introduced to induce luminescence. The emitted light intensity was measured using a photomultiplier tube to determine β-EP concentration. Quality control was performed using Siemens High Abnormal Control, with a CV <8%.

Coagulation parameter analysis: An automated coagulation analyzer (Mindray C2000-A) was employed to assess Activated Partial Thromboplastin Time (APTT), Prothrombin Time (PT), Fibrinogen (FIB), and D-Dimer (D-D) levels. Blood samples were directly analyzed via computer interface. Quality control included daily testing high and low Siemens Coagulation Control samples, with an acceptable International Normalized Ratio (INR) error margin of <5%.

The freeze-thaw cycles of all blood samples were strictly controlled at S3 times to avoid the degradation of cytokines caused by repeated freezing and thawing.

### Statistical methods

Data analysis was performed using GraphPad Prism 9.3. Categorical variables [n(%)] were compared using the chi-square test. For normally distributed continuous data (χ̄±s), independent or paired t-tests were applied as appropriate; non-normally distributed data [median (IQR)] were analyzed using the Mann-Whitney U test or the Wilcoxon signed-rank test. Data distribution was assessed using the Shapiro-Wilk test. A significance level of P<0.05 was adopted for all tests.

## Results

### Baseline data of the subjects were collected

As illustrated in Table 1, the baseline characteristics of both patient groups showed no significant differences (P>0.05), ensuring comparability.

**Table 1:** Clinical data of the two groups of subjects.

Projects		Conventional group	MSK-US group	t (χ^2^)	P
Gender	male	34 (70.83%)	35 (67.31%)	0.145	0.703
	female	14 (29.17%)	17 (32.69%)		
Age (years)		65.56±4.16	66.38±5.52	0.836	0.405
Body Mass Index (kg/m^2^)		24.17±1.26	23.80±1.22	1.465	0.146
Duration (months)		8.40±2.28	8.35±2.04	0.115	0.909
Location of the lesion	L4-L5	26 (54.17%)	25 (48.08%)	0.370	0.543
	L5-S1	22 (45.83%)	27 (51.92%)		
Long-term smoking	yes	28 (58.33%)	26 (50.00%)	0.698	0.404
	no	20 (41.67%)	26 (50.00%)		
Long term drinking	yes	17 (35.42%)	20 (38.46%)	0.099	0.753
	no	31 (64.58%)	32 (61.54%)		
Coexisting chronic diseases	hypertension	26 (54.17%)	26 (50.00%)	0.174	0.677
	diabetes mellitus	21 (43.75%)	25 (48.08%)	0.188	0.665
	hyperlipidemia	14 (29.17%)	18 (34.62%)	0.341	0.560
Habit of exercise	yes	16 (33.33%)	15 (28.85%)	0.235	0.628
	no	32 (66.67%)	37 (71.15%)		

### Impact of MSK-US-guided needle-knife on pain mediators

**Figure 1:**
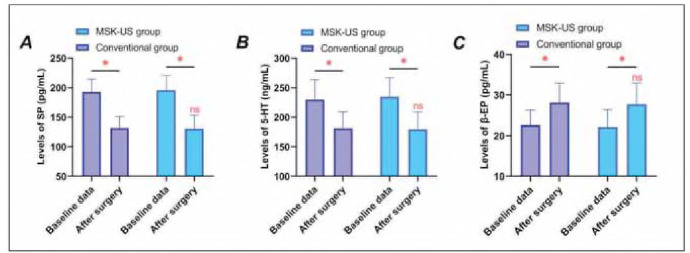
Changes in serum pain mediators after surgery. Panel A: Substance P (SP) levels; Panel B: 5-Hydroxytryptamine (5-HT) levels; Panel C: β-Endorphin (β-EP) levels. * indicates P < 0.05 compared with baseline data, n.s. indicates no statistically significant difference between groups (P < 0.05).

As illustrated in Figure 1, postoperative SP and 5-HT levels in both groups decreased compared to baseline, whereas β-EP levels increased (P<0.05). However, intergroup analysis revealed no significant differences in baseline or postoperative measurements (P>0.05).

### Influence of MSK-US-guided needle-knife on stress response

**Figure 2:**
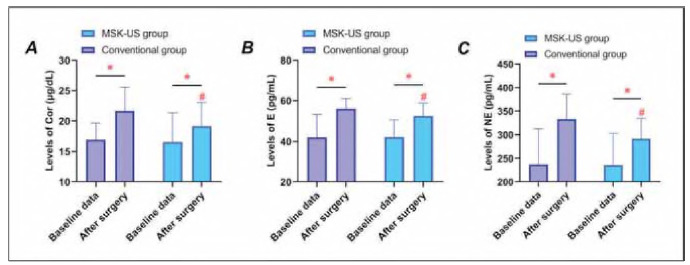
Stress response markers after surgery. Panel A: Cortisol (Cor); Panel B: Epinephrine (E); Panel C: Norepinephrine (NE). * indicates P < 0.05 compared with baseline data, and # indicates P < 0.05 compared with conventional group.

Figure 2 demonstrates that Cor, E, and NE levels rose postoperatively in both groups relative to baseline (P<0.05). Notably, the MSK-US group exhibited lower Cor, E, and NE levels than the conventional group (P<0.05), indicating a milder stress response.

### Influence of MSK-US-guided needle-knife on inflammatory markers

**Figure 3:**
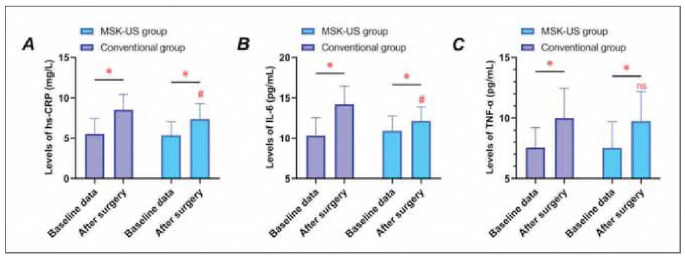
Inflammatory marker dynamics after surgery. Panel A: Interleukin-6 (IL-6); Panel B: Tumor Necrosis Factor-α (TNF-α); Panel C: High-sensitivity C-reactive Protein (hs-CRP). * indicates P < 0.05 compared with baseline data, # indicates P < 0.05 compared with conventional group, n.s. indicates no statistically significant difference between groups (P < 0.05).

Postoperative serum levels of IL-6, TNF-α, and hs-CRP increased in both groups compared to baseline (P<0.05) (Figure 3). While TNF-α did not differ between groups (P>0.05), IL-6 and hs-CRP were significantly reduced in the MSK-US group (P<0.05), suggesting attenuated inflammation.

### Impact of MSK-US-guided needle-knife coagulation parameters

**Figure 4:**
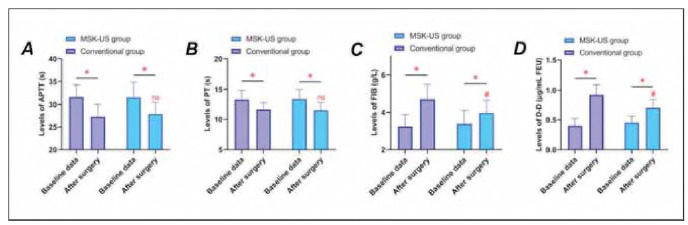
Coagulation function parameters after surgery. Panel A: Activated Partial Thromboplastin Time (APTT); Panel B: Prothrombin Time (PT); Panel C: Fibrinogen (FIB); Panel D: D-Dimer (D-D). * indicates P < 0.05 compared with baseline data, # indicates P < 0.05 compared with conventional group, n.s. indicates no statistically significant difference between groups (P < 0.05).

As depicted in Figure 4, APTT and PT shortened postoperatively in both groups (P<0.05), though remaining within normal limits. D-D and FIB levels increased post-surgery compared to baseline, with higher values observed in the conventional group versus the MSK-US group (P<0.05). These findings imply greater coagulation system activation in the conventional group compared to the MSK-US group.

## Discussion

**Figure 5:**
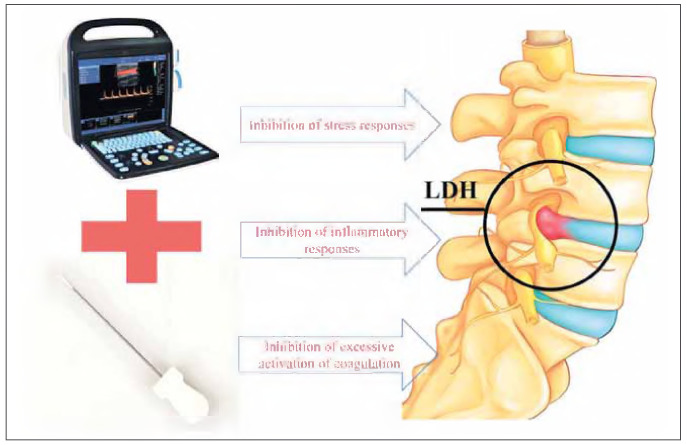
Mechanistic model of MSK-US-guided needle-knife therapy in LDH. Precision Release: Ultrasound guidance minimizes tissue damage and adhesion lysis. Anti-Inflammatory Effect: Reduced IL-6/hs-CRP and preserved β-EP signaling. Stress Attenuation: Lower Cor/E/NE levels via reduced tissue trauma. Coagulation Balance: Decreased D-D/FIB activation through minimized vascular injury.

This research innovatively integrates MSK-US imaging with needle-knife therapy, with longitudinal monitoring demonstrating marked alleviation in postoperative stress and inflammation, as well as obvious enhancement in coagulation parameters among LDH patients (we try to reveal this process with Figure 5). These findings indicate that ultrasound-assisted navigation enhances procedural precision while ensuring surgical safety, thereby contributing valuable evidence for developing personalized therapeutic assessment protocols utilizing biomarker analysis. The clinical implications of this research include: Demonstrating that MSK-US guided procedures can minimize iatrogenic trauma to adjacent tissues while lowering perioperative stress reactions and systemic inflammation;

Indicating that pain alleviation might be associated with rapid modulation of central sensitization, providing insights for future investigations into analgesic mechanisms; suggesting that this technique could potentially decrease postoperative complications like deep vein thrombosis via coagulation function preservation, highlighting its significant clinical transformation value [9].

First of all, in the analysis of pain mediators, both groups showed decreased SP and 5-HT while elevated β-EP post-surgery. The reason is that needle-knife alleviates mechanical compression by releasing adhesions surrounding the nerve root, thereby interrupting pain signal transmission [10]. As nerve root irritation is reduced, the production of pain-related mediators like SP and 5-HT declines rapidly after the procedure, leading to a notable drop in their serum levels compared to preoperative measurements. Concurrently, pain relief activates the endogenous analgesic system, causing a temporary rise in β-EP levels, which may reflect a central analgesic response [11]. This study found no significant inter-group difference in serum pain mediator concentrations. This is likely attributed to the less tissue damage by the minimally invasive technique and the better postoperative experiences in patients undergoing this approach than those receiving traditional open surgery, resulting in minimal pain. However, recovery after surgery depends not only on pain control but also on other physiological factors that require close monitoring.

Although surgical treatment activated stress and inflammatory reactions in both groups, the intensity was milder in the MSK-US group, highlighting the advantages of MSK-US-guided needle-knife in reducing pathological damage. Surgical stress responses are essentially an adaptive defense process of the body against tissue injury, with its intensity positively correlated with the damage extent [12]. The observed differences between MSK-US and the conventional approach may be attributed to several factors: ① The ultrasound-guided »point-to-point« release technique minimizes traction damage to healthy tissues and reduces the transmission of harmful signals to the central nervous system [13]. ② Real-time visualization during the procedure eliminates the need for repeated punctures and exploratory maneuvers, thereby decreasing operative time. Intraoperative ultrasound imaging provides anatomical feedback, which may improve the surgeon's confidence in successful operation, thus lowering patients' psychological stress. IL-6 and hs-CRP, key markers of acute inflammation, indicate the equilibrium between tissue damage and recovery [14] [15]. By limiting vascular injury, MSK-US may suppress local inflammatory mediator release, thereby mitigating the cascade of inflammation triggered by the extrinsic coagulation pathway [16]. Furthermore, the reduced tissue trauma associated with precise ultrasound guidance might attenuate the activation of key inflammatory signaling pathways, such as the Toll-like receptor 4/Nuclear factor-kappa B (TLR4/NF-κB) pathway and the Mitogen-activated protein kinase (MAPK) pathway, which are known to be pivotal in driving the production of pro-inflammatory cytokines like IL-6 and TNF-α in response to tissue injury and stress [17]. Future studies measuring pathway-specific markers (e.g., phosphorylated NF-κB p65, p38 MAPK) in serum or local tissues are warranted to validate this hypothesis. Additionally, targeted release techniques may prevent secondary leakage of nucleus pulposus material and the prolonged dispersion of inflammatory agents [18]. Notably, TNF-α levels showed no significant variation between the groups, implying that MSK-US might exert its effects primarily through modulation of downstream inflammatory pathways. However, further investigation is required to validate this hypothesis.

Certainly, alterations in coagulation function are also a critical aspect of postoperative recovery, in addition to inflammatory responses and stress reactions. Maintaining coagulation-fibrinolysis equilibrium is recognized as a pivotal factor in preventing and managing postoperative complications [19]. The needle-knife puncture procedure induces localized capillary damage, triggering coagulation system activation, platelet aggregation, and increased fibrinogen-to-fibrin conversion, leading to a temporary rise in D-D and FIB levels [20]. However, the MSK-US group exhibited lower postoperative D-D and FIB levels, implying that moderate fibrinolysis activation may help suppress inflammatory cell chemotaxis and migration by resolving microthrombi. This phenomenon can be attributed to two factors: first, ultrasound guidance minimizes vascular punctures, thereby reducing the likelihood of microthrombus formation; second, precise tissue release decreases surgical site bleeding, preventing excessive activation of blood components.

Several constraints should be noted in this research: ① Potential geographical bias exists due to a single sample source, requiring expanded participant recruitment from multiple regions. ② The short observation period (2 hours post-surgery) restricts analysis of prolonged treatment effects and biomarker changes; thus extended monitoring is needed. ③ The absence of intraoperative data collection (e.g., total operative time, number of needle insertion attempts, estimated blood loss at the puncture site) limits the ability to quantitatively compare the procedural efficiency and immediate tissue trauma between the two techniques. ④ The absence of molecular/histological confirmation (e.g., MAPK pathway verification via Western blot) calls for deeper mechanistic exploration in subsequent experiments. Further studies will address these gaps comprehensively. ⑤ The absence of a comparison with other established minimally invasive treatments for LDH (e.g., epidural steroid injections, radiofrequency ablation) limits our ability to definitively position the relative efficacy and advantages of MSK-US-guided needle-knife within the broader therapeutic landscape. Future comparative effectiveness studies are needed.

## Conclusion

MSK-US guidance optimizes the biological effect of needle-knife for LDH: while maintaining effective pain relief, it mitigates perioperative stress, inflammation, and coagulation activation risks. These findings not only provide evidence-based medical support for the clinical promotion of ultrasound-guided techniques, but also lay a crucial foundation for developing a full-chain diagnostic and therapeutic system encompassing precision intervention, biomarker modulation, and long-term outcome assessment.

## Dodatak

### Availability of data and materials

The data that support the findings of this study are available from the corresponding author upon reasonable request.

### Funding

Not applicable.

### Acknowledgements

Not applicable.

### Author contributions

XYL. conceived and designed the study, JQ.W. and M.C. wrote and revised the manuscript, WL.L. and JM.J. collected the date, SH.C. analyzed the data,TY.L. visualisation the data and supervised the study, JQ.W. and M.C. made equal contributions in this work as co-first authors. All authors read and approved the final submitted manuscript.

### Conflict of interest statement

All the authors declare that they have no conflict of interest in this work.
